# Editorial: Advancing neurosonology: integrating ultrasound applications and artificial intelligence in neurology

**DOI:** 10.3389/fneur.2026.1858392

**Published:** 2026-05-18

**Authors:** Giuseppe Miceli, Vassilios Tsitouras, Rita Bella, Alder Casadei, Rossana Tassi

**Affiliations:** 1 Department of Health Promotion, Mother and Child Care, Internal Medicine and Medical Specialties (ProMISE), Palermo University, Palermo, Italy; 2 Second Department of Neurosurgery, Aristotle University School of Medicine, Thessaloniki, Greece; 3 Department of Medical and Surgical Sciences and Advanced Technologies “G. F. Ingrassia”, University of Catania, Catania, Italy; 4 Ultrasound School Società Italiana di Utrasonologia in Medicina e Biologia (SIUMB), Italian Society of Ultrasound in Medicine and Biology, Bolzano, Italy; 5 Stroke Unit, Department of Emergency Urgency, Azienda Ospedaliera Universitaria Senese, Siena, Italy

**Keywords:** artificial intelligence, cerebral flow, neurosonology, transcranial Doppler, ultrasound technology

Neurosonology has emerged as a dynamic and transformative discipline at the intersection of ultrasound technology and clinical neurology (1). Over the past decade, rapid technological advancements, coupled with the integration of artificial intelligence (AI), have significantly expanded its diagnostic and therapeutic horizons. Once considered a primarily operator-dependent bedside tool, neurosonology is now evolving into a sophisticated, multimodal platform capable of providing real-time insights into cerebral hemodynamics, structural integrity, and functional brain responses (2). This Research Topic was conceived to capture these advancements and foster a comprehensive, forward-looking perspective on how ultrasound-based technologies, increasingly augmented by AI, are reshaping neurological care.

The contributions collected in this issue reflect both the breadth and depth of contemporary neurosonology, spanning fundamental pathophysiological insights, methodological standardization, clinical applications, and emerging therapeutic paradigms. Importantly, they highlight a central theme: the transition of neurosonology from a supportive diagnostic modality to a pivotal component of precision neurology.

A key area of advancement lies in refining cerebrovascular diagnostics and pathophysiological understanding. One of the original research articles included in this Research Topic challenges the traditional paradigm that cerebral hypoperfusion in vasovagal syncope (VVS) is solely attributable to systemic hypotension (Hou et al.). By integrating transcranial Doppler (TCD) monitoring with head-up tilt testing, the study identifies a substantial subgroup of patients characterized by impaired cerebral autoregulation despite relatively preserved systemic blood pressure. This distinction between autoregulatory dysfunction and blood pressure–driven mechanisms not only deepens our understanding of VVS pathogenesis but also underscores the critical role of neurosonology in disentangling complex cardiocerebral interactions. Such insights pave the way for more tailored diagnostic and therapeutic approaches, moving beyond conventional hemodynamic models.

The growing importance of neurosonology in cerebrovascular risk stratification is further exemplified by the review on microembolic signal (MES) detection (Sassos et al.). MES monitoring through TCD offers a unique, real-time window into ongoing embolic phenomena, with established relevance in conditions such as large-artery atherosclerosis, atrial fibrillation, and cryptogenic stroke. However, traditional TCD approaches are constrained by operator dependence and limited signal-discrimination capabilities. The integration of AI-driven algorithms and robotic-assisted systems represents a paradigm shift, enabling automated detection, improved artifact rejection, and more nuanced embolus characterization. This convergence of ultrasound and machine learning exemplifies how technological innovation can enhance both diagnostic accuracy and reproducibility, ultimately contributing to more effective secondary prevention strategies.

Complementing these advances, a systematic review and meta-analysis included in this Topic provides a rigorous evaluation of the diagnostic performance of transcranial ultrasound techniques in acute stroke (Pinto-Villalba et al.). By synthesizing evidence from multiple studies, the analysis demonstrates that TCD and transcranial color-coded duplex sonography (TCCS) achieve good sensitivity and specificity for differentiating ischemic from hemorrhagic stroke. While acknowledging inherent limitations, such as acoustic window dependence and operator variability, the findings reinforce the potential of neurosonology as a valuable diagnostic alternative, particularly in resource-limited settings where access to advanced neuroimaging may be limited. This work highlights an important dimension of neurosonology: its capacity to democratize neurological diagnostics through portable, cost-effective technologies.

Standardization remains a critical challenge for the broader clinical integration of neurosonology. Addressing this need, the position statement developed under the auspices of the Italian Society of Neurosonology and Cerebral Hemodynamics (Miceli et al.) provides consensus-based recommendations on the use of contrast-enhanced TCD for detecting right-to-left shunts, particularly in the context of patent foramen ovale (PFO). Through a structured literature review and a Delphi consensus process involving multiple expert centers, the statement establishes a unified protocol for examination execution, interpretation, and reporting. Such efforts are essential to reduce inter-operator variability, enhance diagnostic reliability, and facilitate multicenter research collaborations. In this regard, the article serves as a model of how methodological rigor and expert consensus can accelerate the translation of neurosonological techniques into routine clinical practice.

Beyond cerebrovascular diagnostics, neurosonology is increasingly contributing to the management of critically ill neurological patients. The study on optic nerve sheath diameter (ONSD) ultrasonography in pediatric intracranial hypertension illustrates the value of bedside ultrasound as a non-invasive monitoring tool (Hua et al.). By enabling dynamic assessment of intracranial pressure surrogates, ONSD measurement allows for more responsive and individualized therapeutic interventions. The reported improvements in neurological outcomes and treatment optimization underscore the clinical utility of integrating ultrasound into neurocritical care protocols. This application exemplifies the broader trend toward multimodal, non-invasive monitoring strategies that enhance patient safety while providing actionable clinical data.

Finally, the expanding therapeutic potential of neurosonology is highlighted by the systematic review on transcranial ultrasound stimulation (TUS) (Wang et al.). As a non-invasive neuromodulation technique, TUS offers a novel avenue to influence brain function with high spatial precision. The analysis of stimulation parameters and their physiological effects provides critical insights into dose–response relationships and safety profiles, laying the groundwork for standardized protocols. The observed disease-specific effects, ranging from modulation of neuroplasticity in neurodegenerative disorders to improvement of motor function, underscore the versatility of ultrasound-based neuromodulation. However, the authors appropriately emphasize the need for large-scale, well-designed clinical trials to validate these findings and facilitate clinical translation.

Taken together, the articles in this Research Topic illustrate a field in rapid evolution, characterized by increasing technological sophistication and expanding clinical relevance. Several overarching themes emerge. First, neurosonology is becoming an indispensable tool for real-time, bedside assessment of neurological function, bridging the gap between structural imaging and physiological monitoring (2). Second, the integration of AI is transforming data acquisition and interpretation, addressing longstanding limitations related to operator dependency and reproducibility (3). Third, standardization and consensus-building efforts are essential to ensure the reliability and scalability of neurosonological techniques across diverse clinical settings. Finally, the therapeutic applications of ultrasound, particularly in neuromodulation, represent a frontier with profound implications for the future of neurology.

Despite these advances, significant challenges remain. The full integration of neurosonology into routine clinical workflows requires not only technological innovation but also structured training programs, validation studies, and interdisciplinary collaboration. Moreover, ethical considerations related to AI implementation, data governance, and patient safety must be carefully addressed.

In conclusion, this Research Topic provides a comprehensive overview of the current state and future directions of neurosonology. By bringing together diverse yet complementary contributions, it underscores the transformative potential of combining advanced ultrasound technologies with artificial intelligence. As the field continues to evolve, neurosonology is poised to play a central role in advancing precision neurology, ultimately improving diagnostic accuracy, therapeutic efficacy, and patient outcomes.
